# Gene expression variation in *Arabidopsis* embryos at single-nucleus resolution

**DOI:** 10.1242/dev.199589

**Published:** 2021-07-06

**Authors:** Ping Kao, Michael A. Schon, Magdalena Mosiolek, Balaji Enugutti, Michael D. Nodine

**Affiliations:** 1 Gregor Mendel Institute (GMI), Austrian Academy of Sciences, Vienna Bio Center (VBC), Dr Bohr-Gasse 3, 1030 Vienna, Austria; 2 Laboratory of Molecular Biology, Wageningen University, Wageningen 6708 PB, The Netherlands

**Keywords:** *Arabidopsis*, Embryo, Single-nucleus RNA-seq, Gene expression, Transcription factor, Epigenetic

## Abstract

Soon after fertilization of egg and sperm, plant genomes become transcriptionally activated and drive a series of coordinated cell divisions to form the basic body plan during embryogenesis. Early embryonic cells rapidly diversify from each other, and investigation of the corresponding gene expression dynamics can help elucidate underlying cellular differentiation programs. However, current plant embryonic transcriptome datasets either lack cell-specific information or have RNA contamination from surrounding non-embryonic tissues**.** We have coupled fluorescence-activated nuclei sorting together with single-nucleus mRNA-sequencing to construct a gene expression atlas of *Arabidopsis thaliana* early embryos at single-cell resolution. In addition to characterizing cell-specific transcriptomes, we found evidence that distinct epigenetic and transcriptional regulatory mechanisms operate across emerging embryonic cell types. These datasets and analyses, as well as the approach we devised, are expected to facilitate the discovery of molecular mechanisms underlying pattern formation in plant embryos.

This article has an associated ‘The people behind the papers’ interview.

## Abstract (precis)

**Highlighted Article:** A transcriptome atlas of *Arabidopsis* embryos constructed from single nuclei reveals cell-specific epigenetic and transcriptional regulatory features.

## INTRODUCTION

Metazoans and land plants establish their body plans during embryogenesis (Dresselhaus and Jürgens, 2021; Gerri et al., 2020), and corresponding gene regulatory mechanisms have evolved independently in these two major eukaryotic lineages to help generate the immense morphological diversity observed in nature (Bai, 2015; Clark et al., 2006; Meyerowitz, 2002). For example, in animals it has been long recognized that maternal gene products control initial pattern formation before the transition of control from the maternal to the zygotic genome (Lee et al., 2014; Tadros and Lipshitz, 2009). By contrast, transcriptional activation of the zygotic genome soon after fertilization is necessary for zygote elongation and initial divisions in *Nicotiana tabacum* (tobacco) (Zhao et al., 2011) and the model flowering plant *Arabidopsis thaliana* (*Arabidopsis*) (Kao and Nodine, 2019; Zhao et al., 2019). In addition, the vast majority of genes regulating *Arabidopsis* embryo morphogenesis are zygotically expressed (Nodine and Bartel, 2012; Zhao et al., 2019) and required (Meinke, 2020; Muralla et al., 2011). Therefore, genes are expressed from the zygotic genome during initial stages of embryo development, and the diversification of gene expression programs across plant embryonic cell types contributes to the formation of the basic body plan. Characterizing how gene expression programs are established in individual cell types of early embryos is crucial to understand the molecular basis of pattern formation in plant embryos, and more broadly the general and unique principles of embryonic patterning in multicellular organisms.

Forward genetic screens successfully identified many genes that are required for proper plant embryogenesis (Lukowitz et al., 2004; Mayer et al., 1998; Meinke, 2020), but relatively few mutations in genes encoding cell-specific transcriptional regulators were recovered. This is at least partially due to the high degree of genetic redundancy among plant transcription factors (TFs) that typically belong to multigene families (Riechmann, 2002). As an alternative approach, RNA populations can be characterized to infer gene-regulatory processes underlying cellular differentiation events. Transcriptomes generated from early embryos at various stages of development have accordingly yielded insights into the biological processes operating during different embryonic phases (Belmonte et al., 2013; Hofmann et al., 2019; Xiang et al., 2011; Zhao et al., 2019). However, these transcriptomes were generated from whole embryos. Additional studies have revealed genes that are preferentially expressed in broad (Belmonte et al., 2013; Casson et al., 2005; Chen et al., 2021; Slane et al., 2014; Zhou et al., 2020) or more specific (Palovaara et al., 2017) regions of plant embryos, but either lack cellular resolution or were contaminated with RNAs derived from the maternal seed coat that encompasses the developing embryo (Schon and Nodine, 2017).

Single-cell mRNA-sequencing (scRNA-seq) has been instrumental towards understanding developmental events at cellular resolution over the past decade (Chen et al., 2019; Hwang et al., 2018). Several studies have applied these approaches to plant tissues (Brennecke et al., 2013; Efroni and Birnbaum, 2016; Jean-Baptiste et al., 2019; Ryu et al., 2019; Satterlee et al., 2020; Shulse et al., 2019; Song et al., 2020; Xu et al., 2021; Zhang et al., 2019), but scRNA-seq has yet to be reported for individual cell types in plant embryos. This is primarily due to the presence of rigid cell walls that hold plant cells together. Although cell walls can be removed by enzymatic treatment of tissues that are easy to access, such protoplasting techniques remain impractical for early embryos because they are deeply embedded within maternal seed tissues. Single-nucleus mRNA-sequencing (snRNA-seq) (Habib et al., 2016) offers an alternative method to inspect transcriptomes at single-cell resolution in plants and has been recently applied to roots (Farmer et al., 2021) and endosperm tissues within seeds (Long et al., 2021; Picard et al., 2021). Here, we present a workflow to obtain contamination-free high-quality transcriptomes from individual early embryonic nuclei followed by their assignments to the precursors of the most fundamental plant tissues including the shoot meristem, distal regions of the root meristem and epidermal and vascular tissues. Remarkably, these initial embryonic cell types already express characteristic sets of genes, have different evolutionary trajectories and appear to be regulated by distinct epigenetic and transcriptional mechanisms.

## RESULTS

### Acquisition of contamination-free transcriptomes from individual embryonic nuclei

**Fig. 1.**
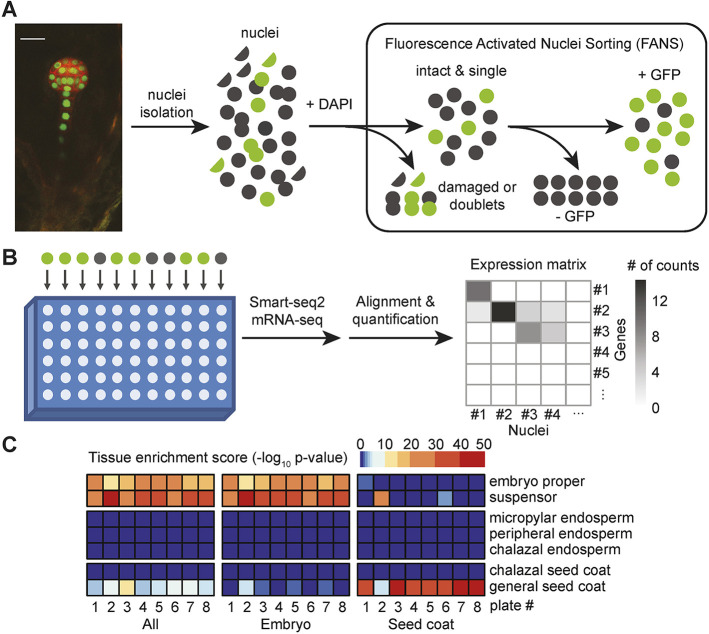
**Acquisition of contamination-free transcriptomes from individual embryonic nuclei.** (A) Schematic showing collection of single embryonic nuclei. GFP-positive fixed nuclei from pWOX2::NLS-GFP transgenic developing seeds were sorted and collected by FANS. Scale bar: 20 μm. (B) Diagram showing how single-nucleus libraries were generated with a modified Smart-seq2 protocol (Picelli et al., 2014a,b), sequenced and individual gene expression quantified. (C) Maternal contamination assessment and removal. Nuclei from each plate were assigned as embryonic or seed-coat-derived according to the unsupervised clustering and tissue enrichment tests (Fig. S2A-C). Tissue enrichment tests based on the mean expression of all nuclei or nuclei categorized as embryo or seed coat are shown.

To acquire single-cell transcriptomes of early *Arabidopsis* embryos, we used fluorescence-activated nuclei sorting (FANS) coupled with snRNA-seq (Fig. 1A,B). More specifically, we used a transgenic line expressing nuclear-localized green fluorescent protein (GFP) under the control of the embryo-specific *WUSCHEL-RELATED HOMEOBOX 2* (*WOX2*) promoter (pWOX2::H2B-GFP, pWOX2::tdTomato-LTI6b; hereafter referred to as pWOX2::NLS-GFP) to fluorescently label nuclei in embryos but not the surrounding endosperm or maternal tissues (Fig. 1A) (Gooh et al., 2015). We chose to focus on globular-stage embryos because this is when the precursors to the most fundamental plant tissues emerge along apical-basal and radial embryonic axes (Palovaara et al., 2016). Briefly, we fixed siliques or seeds containing globular-stage embryos with a low concentration of dithiobis (succinimidyl propionate) (DSP) before nuclei isolation to preserve RNA. Nuclei were also stained with 4′,6-diamidino-2-phenylindole (DAPI), and intact nuclei were selected based on DAPI profiles (Fig. S1A,C). Embryonic nuclei were then isolated based on their strong GFP signal (Fig. S1B,D) and sorted individually into 96-well plates. Fixed nuclei were decrosslinked with dithiothreitol (DTT) to enable the generation of cDNA and the Smart-seq2 protocol (Picelli et al., 2014a,b) was used to construct next-generation sequencing (NGS)-compatible libraries. NGS libraries were then sequenced on an Illumina HiSeq 2500 followed by the alignment of NGS reads to the Araport11 transcriptome (Cheng et al., 2017) and transcript quantification by Kallisto (Bray et al., 2016) (Fig. 1B). After quality controls (see Materials and Methods), 534 out of 744 (72%) nuclei were retained for further analyses. A total of 24,591 genes were detected from all nuclei with an average of 440,289 aligned reads and 2576 detected genes per snRNA-seq library (Fig. S1E,F; Table S1). Therefore, our approach allowed us to acquire high-quality RNA-seq libraries from hundreds of individual embryonic nuclei.

Contamination of early embryonic mRNA-seq datasets with RNAs from surrounding maternal seed tissues has been a major limitation to embryo transcriptomics (Schon and Nodine, 2017). To evaluate the level of maternal contamination in individual snRNA-seq libraries, we applied the tissue enrichment test (Schon and Nodine, 2017). Although we attempted to achieve 99.9% accuracy with our stringent FANS selection (Fig. S1), embryonic nuclei comprised only 0.1-1% of seed nuclei and thus false positive events were non-negligible and further filtering was required. Accordingly, 20-50% of the snRNA-seq libraries per plate were significantly enriched for either seed coat or endosperm transcripts, whereas remaining snRNA-seq libraries were enriched for embryonic transcripts or had ambiguous identities (Fig. S2A). To systematically identify contaminated snRNA-seq libraries, we conducted unsupervised clustering on all libraries and labeled them according to their tissue enrichment scores (Fig. S2B,C). Because clusters 12 and 13 were enriched for libraries with seed coat contamination, we excluded them from subsequent analyses (Fig. S2B,C). To further evaluate how well we could remove non-embryonic nuclei, we combined the expression levels of snRNA-seq libraries from each plate and performed tissue enrichment tests (Fig. 1C). Retained and excluded nuclei were enriched for embryonic and seed coat cell types, respectively. Moreover, transcriptomes from the retained nuclei were more similar to published embryonic transcriptomes (Hofmann et al., 2019; Nodine and Bartel, 2012) than those from discarded nuclei (Fig. S2C). Altogether, our stringent criteria allowed us to successfully remove non-embryonic snRNA-seq libraries and obtain 486 high-quality snRNA-seq libraries from embryonic cells.

### Identification of embryonic cell types

**Fig. 2.**
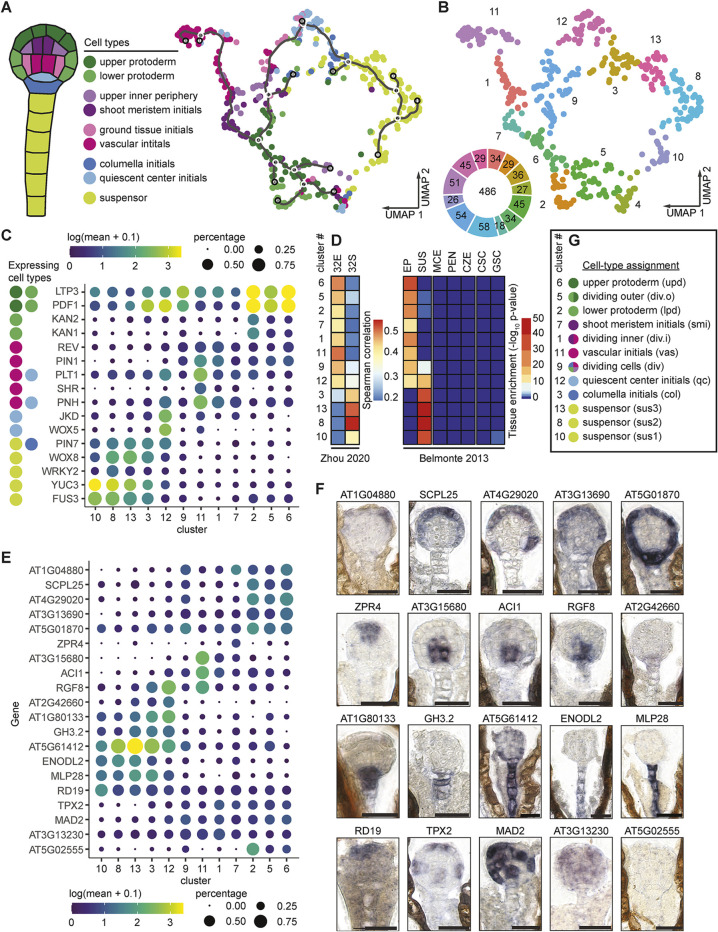
**Identification of embryonic cell types.** (A) Resolving nine defined cell types by supervised clustering. Marker genes expressed in at least one of the nine cell types were used to calculate cell-type scores with hypergeometric tests. Each dot represents a nucleus and nuclei were labeled according to the cell type with the highest cell-type score. (B) The thirteen clusters corresponding to A. The number of nuclei for each cluster is indicated in the donut plot. (C) Dot plot illustrating expression patterns of known cell type-specific markers. The cell types in which a marker gene is expressed were color-coded according to A. The sizes of dots represent the percentage of nuclei in which the transcript was detected for each cluster and the colors represent the log_10_-transformed mean expression levels of each cluster. (D) Spearman's correlation coefficients between cluster mean expression and published globular-stage embryo proper (32E) and suspensor (32S) transcriptomes (Zhou et al., 2020) (left). Tissue enrichment test results based on cluster mean expression using published transcriptomes from seed tissues as a reference (Belmonte et al., 2013; Schon and Nodine, 2017) (right). EP, embryo proper; SUS, suspensor; MCE, micropylar endosperm; PEN, peripheral endosperm; CZE, chalazal endosperm; CSC, chalazal seed coat; GSC, general seed coat. (E) Dot plot of expression patterns of transcripts selected for RNA ISH as in C. Dot plots for the remaining 13 RNA ISH candidates are shown in Fig. S3E. (F) Representative RNA ISH images for 20 selected transcripts. The remaining 13 RNA ISH candidates are presented in Fig. S3F. Scale bars: 20 μm. Quantification of RNA ISH images is shown in Fig. S4. (G) Assigned cell types and abbreviations for clusters.

Unsupervised uniform manifold approximation and projection (UMAP) clustering of snRNA-seq libraries was able to distinguish embryo proper and suspensor nuclei, but not individual cell types (Fig. S3A). The inability of unsupervised UMAP clustering to resolve individual cell types could be due to the relatively low number of snRNA-seq libraries used (*n*=486) or the transient fates of early embryonic cell types. Nevertheless, as an alternative to unsupervised clustering we used enrichments and depletions of known cell-specific transcripts in each nucleus to determine how likely it was for each nucleus to come from each cell type. We first identified 174 reference genes expressed in embryos from the literature and recorded their expression patterns as either expressed or not expressed in the cell types present in globular embryos (Table S2; Fig. 2A). More specifically, nine cell types are found in globular embryos (Palovaara et al., 2016) and can be classified based on whether they derive from the larger basal cell or smaller apical cell formed upon zygote division. The corresponding basal cell lineage (BCL) consists of the terminally differentiated suspensor, which connects maternal tissues with the embryo proper, as well as the columella and quiescent center initials, which are precursors to distal regions of the root meristem. Unlike the BCL, the apical cell lineage (ACL) divides along the radial embryonic axis to form concentric tissue layers. The outermost protoderm, middle ground tissue initials and innermost vascular initials produce the epidermal, ground and vascular tissues, respectively; whereas the shoot meristem initials will produce aerial tissues after germination. Presence or absence of these reference genes were then used in hypergeometric tests to compute cell-type scores for each nucleus of these nine cell types in all 486 snRNA-seq libraries (see Materials and Methods). We then performed UMAP clustering on the cell-type scores, and identified 12 clusters that were each enriched for a specific cell type (Fig. 2A,B). We also identified one cluster (cluster 4) that was enriched for multiple cell types and had substantially fewer genes detected per snRNA-seq library compared with the other clusters. We discarded the snRNA-seq libraries belonging to this cluster from subsequent analyses because of their poor quality, which may be due to being generated from aggregated or fragmented nuclei. Cell-specific reference transcripts tended to co-localize to the same cluster (Fig. 2C; Fig. S3B) indicating that clustering on cell-type scores recapitulates expression patterns of reference markers. For example, WOX5 and JACKDAW (JKD) transcripts are highly enriched in the quiescent center initials (Haecker et al., 2004; Welch et al., 2007) and co-localize to cluster 12. Therefore, by highlighting the differences among cell types based on a reference gene set we were able to resolve the 486 snRNA-seq libraries into 12 clusters representing distinct cell types.

To independently test these marker-based predictions, we compared the transcriptomes of each cell cluster with published transcriptomes from the embryo proper and suspensor regions of globular embryos (Belmonte et al., 2013). In agreement with the marker-based assignments, clusters 8, 10 and 13 were exclusively enriched for suspensor transcripts based on tissue enrichment tests (Fig. 2D). Also consistent with the cell type assignments, clusters 1, 2, 5, 6, 7 and 11 were enriched for only embryo proper transcripts. Cluster 9 had mixed cell type assignments and accordingly was enriched for both embryo proper and suspensor transcripts. Most of the nuclei in clusters 3 and 12 were, respectively, labeled as columella initials and quiescent center initials, which are situated between the suspensor and embryo proper. Whereas cluster 3 was only enriched for suspensor transcripts, cluster 12 was enriched for both suspensor and embryo proper transcripts. We also confirmed these results with another published transcriptome dataset generated from embryo propers and suspensors of globular embryos (Zhou et al., 2020) (Fig. 2D). As further support for the cell type assignments of the clusters, three genes not included in our reference list were recently found to be specifically expressed in vascular initials (Smit et al., 2020) and all three were specific to cluster 11 (Fig. S3C) along with other vascular-expressed genes (Fig. 2C).

We then used RNA *in situ* hybridization (ISH) to further evaluate the marker-based assignments of snRNA-seq clusters to individual cell types. We selected 33 genes without reported expression patterns that represented a specific cell or group of cells based on their expression patterns (Fig. 2E; Fig. S3D). We could detect RNA ISH signal in at least 50% of embryos for 26 of the 33 probes tested (78.8%) and compared the RNA ISH and snRNA-seq expression patterns for these in more detail (Fig. 2F; Fig. S3E,F and Fig. S4). *AT3G13690*, *AT4G29020* and *SERINE CARBOXYPEPTIDASE-LIKE 25* (*SCPL25*) were expressed at high levels in clusters 2, 5 and 6, and detected by RNA ISH almost exclusively in the protoderm. *AT5G01870* was also highly expressed in clusters 2, 5 and 6, as well as cluster 12, and was detected in the protoderm and columella initials; whereas *AT1G04880* was expressed in clusters 6 and 9, and detected in the upper protoderm. *AT1G80133*, *AT2G42660*, *AT3G54780* and *GH3.2* were highly expressed in clusters 3 and 12, and RNA ISH signals were detected in the columella and quiescent center initials, as well as throughout the suspensors for GH3.2. *LITTLE ZIPPER 4* (*ZPR4*) was specifically expressed in cluster 7 based on snRNA-seq and detected in the shoot meristem initials by RNA ISH. RESPONSIVE TO DEHYDRATION 19 (RD19) transcripts were also detected by RNA ISH in shoot meristem initials, but were moderately expressed in all clusters. Similarly, *AT4G38370* was expressed throughout the clusters, albeit most strongly in cluster 8, but the RNA ISH signal was stronger in the embryo proper. These two apparent discrepancies between gene expression and RNA localization may be due to differences between nuclear and cytoplasmic mRNA populations, including variability in post-transcriptional regulation among cell types. *ALCATRAZ-INTERACTING PROTEIN 1* (*ACI1*), *AT3G15680* and *AT3G15720* were expressed most highly in cluster 11 and detected in vascular initials with RNA ISH. *ROOT MERISTEM GROWTH FACTOR 8* (*RGF8*) was highly expressed in clusters 11 and 12, and RGF8 transcripts were detected in vascular and columella initials. *AT5G61412*, *BETA GLUCOSIDASE 17* (*BGLU17*), *COBRA-LIKE PROTEIN 6 PRECURSOR* (*COBL6*), *CYSTEINE ENDOPEPTIDASE 1* (*CEP1*), *EARLY NODULIN-LIKE PROTEIN 2* (*ENODL2*), *MAJOR LATEX PROTEIN 28* (*MLP28*) and *SPERMIDINE DISINAPOYL ACYLTRANSFERASE* (*SDT*) were expressed in clusters 8, 10 or 13, and all their corresponding transcripts were detected in suspensors by RNA ISH. *AT3G13230*, *MITOTIC ARREST-DEFICIENT 2* (*MAD2*) and *TARGETING PROTEIN FOR XKLP2* (*TPX2*) were highly expressed in clusters 1, 5 and/or 9, and corresponding RNA ISH produced ‘salt-and-pepper’ patterns, which are indicative of cell-cycle regulated genes. Accordingly, we observed that clusters 1, 5 and 9 were enriched for mitotic-phase-regulated transcripts (Menges et al., 2003) (Fig. S3G). Genes preferentially expressed in clusters 1, 5 and 9 also tended to be localized to the subprotoderm, protoderm or both layers, respectively. Therefore, our results suggested that clusters 1, 5 and 9 represent dividing subprotoderm (dividing inner; div.i), protoderm (dividing outer; div.o) and dividing cells in general (div), respectively (Fig. 2G). Altogether, our *in silico* and *in situ* validations indicated that we can assign groups of snRNA-seq libraries to the major cell types present in globular embryos: the suspensor (sus1, cluster 10; sus2, cluster 8; sus3, cluster 13); columella initials (col; cluster 3), quiescent center initials (qc; cluster 12); vascular initials (vas; cluster 11); shoot meristem initials (smi; cluster 7); and the lower and upper protoderm (lpd, cluster 2; upd, cluster 6) (Fig. 2G).

### General characteristics of transcriptomes from embryonic cell types

**Fig. 3.**
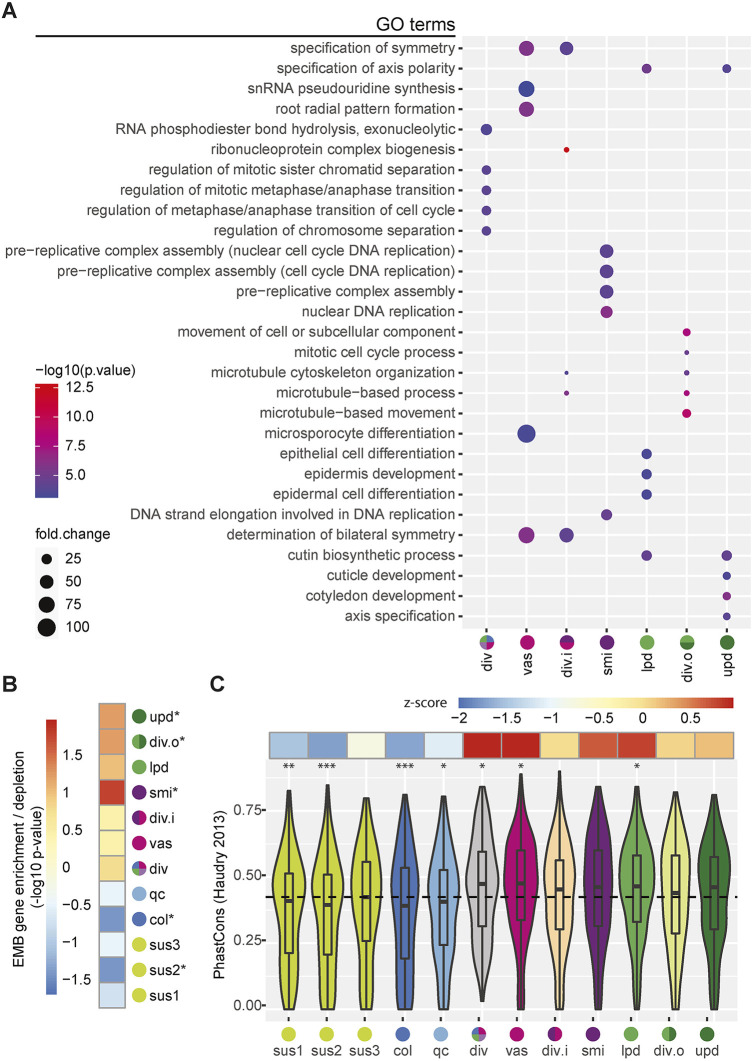
**General characteristics of transcriptomes from embryonic cell types.** (A) The top-five enriched gene ontology (GO) terms identified by PANTHER for each cluster according to the top-250 ranked genes for each cluster. The suspensor clusters (8,10,13) and hypophysis clusters (3,12) did not have significantly enriched GO terms and thus are not shown. The sizes and colors of the dots represent the fold changes and -log_10_-transformed *P*-values, respectively. (B) Levels of overrepresentation of embryo-defective (EMB) genes for the top-250 ranked genes for each cluster. Asterisks indicate significant (*P*≤0.05) enrichment or depletion of EMB genes relative to expectations. (C) PhastCons conservation scores (Haudry et al., 2013) of top-250 ranked genes for each cluster. The mean PhastCons score of all expressed genes is indicated by a dashed line, and deviations from the mean are presented in the upper row as *z*-scores. *P≤0.05, **P≤0.01, ***P≤0.001; based on two-sided Kolmogorov–Smirnov tests with the alternative hypothesis that the cluster conservation score distributions of the top-ranked 250 genes were not equal to that of all expressed genes in embryos. PhastCons and PhyloP scores from another report (Tian et al., 2020) had similar trends (Fig. S5).

To provide a concise and uniform parameter to examine gene expression patterns across embryonic cell types, we calculated ‘enrichment scores’ in each of the 12 clusters for the 13,893 transcripts detected in ≥10% of nuclei within ≥1 cluster (Table S3). Enrichment scores are a combination of the deviations of mean transcript levels and the percentage of nuclei it was detected in for each cluster relative to the other 11 clusters (see Materials and Methods; Table S3), and thus concisely summarize the relative abundance of each transcript in each cluster. The 250 genes with the highest enrichment scores (top-ranked 250) from each cluster were considered preferentially expressed genes for that cluster. Enrichment scores of known markers matched their reported expression patterns (Fig. 2C; Fig. S3D). For example, 74 of the 118 (62.7%) reference genes were within top-ranked 250 genes of at least one cluster, including four that were top-ranked: *PIN-FORMED 1* (*PIN1*; cluster 1), *KANADI 1* (*KAN1*; cluster 2), *WOX5* (cluster 12) and *WUSCHEL RELATED HOMEOBOX 8* (*WOX8*; cluster 13). To gain insights into which biological processes are enriched in each embryonic cell type, we conducted gene ontology (GO) term enrichment analyses on the top-250 ranked genes of each cluster (Fig. 3A; Table S4). Significantly enriched GO terms were identified for the top-ranked 250 genes in the div, vas, div.i, smi, lpd, div.o and upd clusters, but not sus1/2/3, col or qc clusters. The inability to detect enriched terms in these BCL clusters may have been due to the limited annotation of genes specifically expressed in these cell types. Consistent with the div, div.i and div.o clusters representing actively dividing cells, GO terms related to progression through mitotic phases (div and div.o) and microtubules (div.i and div.o) were enriched. GO terms related to body axis specification were also enriched in the top-250 ranked genes of the div.i cluster, as well as the vas cluster. The ‘microsporocyte differentiation’ GO term is only associated with *BARELY ANY MERISTEM 1* (*BAM1*) and *BAM2*, which encode receptor-like kinases. Both were among the top-250 ranked genes of the vas cluster (Table S3) and are required for vascular patterning in leaf and root tissues (DeYoung et al., 2006; Fan et al., 2021). The protoderm clusters (lpd and upd) were both enriched for specification of axis polarity and cutin biosynthesis terms within their top-250 ranked genes. Moreover, the top-250 ranked genes of the lpd and upd clusters could be distinguished from each other by their overrepresentation of epidermal and cotyledon development GO terms, respectively. The top-250 ranked genes of the smi cluster were enriched for genes involved in DNA replication processes, including pre-replicative complex assembly, which is consistent with the smi cluster being depleted for mitosis phase markers (Fig. S3G). Overall, the enriched GO terms were consistent with the assigned cluster identities (Fig. 2G) and indicate that we have classified embryonic cell types with distinct functions.

Next, we tested whether genes essential for embryogenesis are preferentially enriched within the top-250 ranked genes of each cluster. EMBRYO-DEFECTIVE (EMB) genes are a set of genes required for normal embryo development in Arabidopsis (Meinke, 2020). EMB genes were enriched in the top-250 genes of the ACL clusters including significant enrichment in the smi, div.o and upd clusters. By contrast, EMB genes were depleted from top-250 genes of the BCL clusters, including significant depletion in the col and sus2 clusters (Fig. 3B). Further supporting that genes preferentially expressed in the ACL are more likely to be required for proper development than those in the BCL, we found that the top-250 ranked genes within the ACL, and especially the div, vas and lpd clusters, were more highly conserved across Brassicaceae species and land plants in general compared with BCL clusters (Haudry et al., 2013; Tian et al., 2020) (Fig. 3C; Fig. S5A,B). Also consistent with the EMB analyses, the top-250 ranked genes within the BCL clusters were more poorly conserved, especially genes enriched in the sus1, sus2, col and qc clusters. Altogether, these results suggested that genes preferentially expressed in ACL clusters, and especially the vas and div clusters, are under stronger purifying selection compared with those in BCL clusters, especially the col cluster, which are mutating at a faster rate. This is also consistent with the more variable morphologies of suspensors relative to embryo propers (Chen et al., 2021).

### Transcripts encoding epigenetic regulators vary across embryonic cell types

**Fig. 4.**
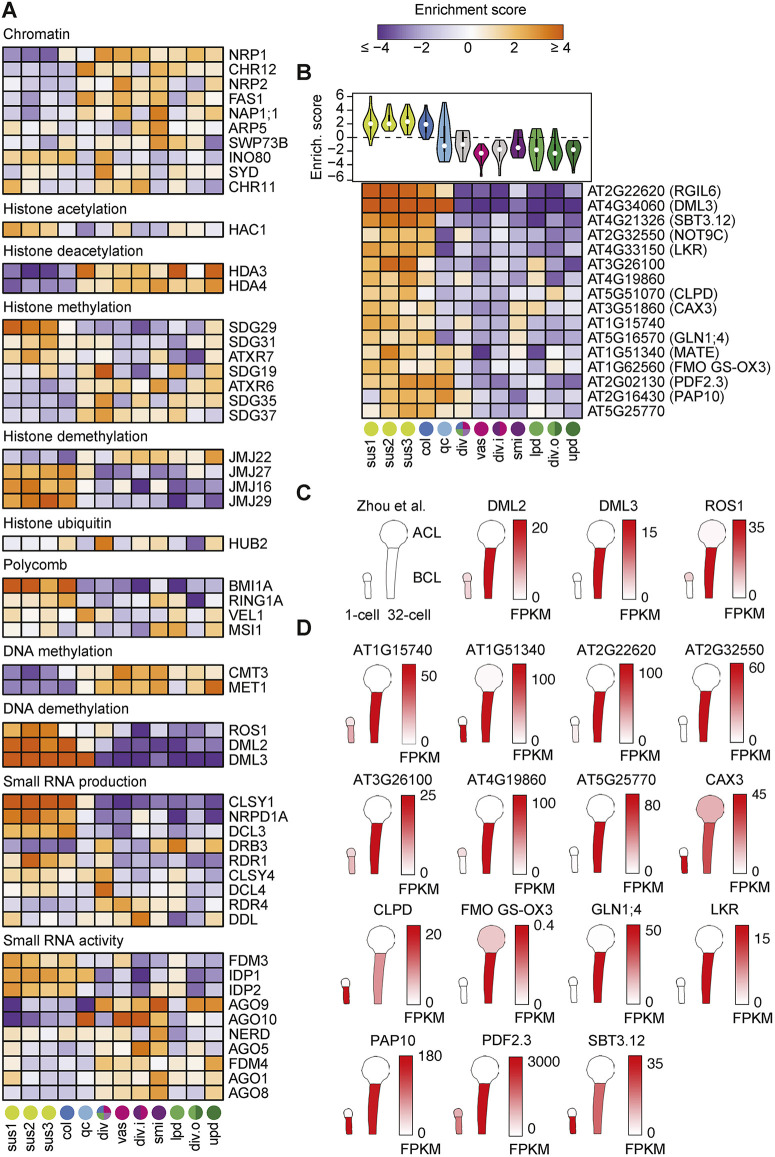
**Transcripts encoding epigenetic regulators vary across embryonic cell types.** (A) Heatmap illustrating enrichment scores in 12 clusters corresponding to different embryonic cell types. Transcripts with enrichment scores ≥2.5 in ≥1 cell cluster are shown and enrichment scores are colored according to key. Gene names are indicated and cluster identities are marked and color-coded at the bottom according to Fig. 2G. (B) Violin plot (top) and heatmap (bottom) of enrichment scores for 16/50 ROS1/DML2/DML3 (RDD) targets detected and enriched in the basal cell lineage. (C,D) Schematic of RDD transcripts (C) and their putative embryonic targets (D) based on published mRNA-seq from apical and basal cell lineages in one-cell and 32-cell stage embryos (Zhou et al., 2020). Transcript levels (fragments per kilobase of transcript per million mapped reads; FPKM) are colored according to the keys.

Soon after fertilization of egg and sperm, epigenetic states are reprogrammed in the new generation (Gehring, 2019). This includes replacement of histones, as well as re-establishment of DNA methylation landscapes genome-wide by small RNA-dependent and -independent pathways (Bouyer et al., 2017; Ingouff et al., 2010; Jullien et al., 2012; Nagasaki et al., 2007; Papareddy et al., 2020). Because such differential chromatin states can strongly influence gene expression, we examined the transcript levels of genes previously implicated in chromatin regulation. More specifically, we found that 50/191 genes involved in general chromatin features, histone modifications (i.e. acetylation, methylation and ubiquitination), polycomb repressive complexes, DNA methylation or demethylation, or small RNA production or activities, had enrichment scores ≥2.5 in ≥1 embryonic cell cluster (Erdmann and Picard, 2020; Pikaard and Mittelsten Scheid, 2014) (Fig. 4A). General chromatin factors and components of the polycomb repressive complex tended to vary between the embryo proper and suspensor. HISTONE ACETYLTRANSFERASE OF THE CBP FAMILY 1 (HAC1) was enriched in the suspensor clusters, whereas HISTONE DEACETYLASE 3/4 (HDA3/4) were enriched in the embryo proper. Moreover, the JUMONJI DOMAIN-CONTAINING16/27/29 (JMJ16/27/29) and JMJ22 histone demethylases were enriched in the suspensor and embryo proper, respectively. Interestingly, the terminally differentiated suspensor was enriched for transcripts encoding proteins required for the production of 24-nt small interfering RNAs (siRNAs) such as CLASSY1 (CLSY1), NUCLEAR RNA POLYMERASE D1A (NRPD1A) and DICER-LIKE3 (DCL3) and this was consistent with previously published datasets (Belmonte et al., 2013; Zhou et al., 2020). By contrast, genes encoding Argonaute (AGO; AGO1/5/8/9/10) proteins, which bind to small RNAs and mediate gene repression, were enriched in the precursors of the shoot meristem initials. The enrichment of AGOs in shoot meristem initials is supported by previous reports (Gutzat et al., 2020; Jullien et al., 2020 preprint; Tucker et al., 2008) and is consistent with small RNA-mediated surveillance pathways that prevent transposon mobilization and other genome de-stabilizing events being enriched in the precursors to all aerial tissues including the gametes. Altogether, these results suggest that small RNA-dependent and -independent pathways establish distinct chromatin environments in individual cell lineage precursors.

The most striking cell-specific enrichments were in pathways affecting cytosine methylation, which is typically associated with transcriptional silencing of transposons and repression of gene promoters (Law and Jacobsen, 2010). CHROMOMETHYLTRANSFERASE 3 (CMT3) and METHYLTRANSFERASE 1 (MET1) encode DNA methyltransferases that maintain cytosine methylation in the CHG (H≠G) and CG contexts, respectively, and both were enriched in the embryo proper. By contrast, transcripts encoding the REPRESSOR OF SILENCING (ROS1), DEMETER-LIKE 2 (DML2) and DML3 DNA glycosylases required for the removal of methylated cytosines were highly enriched in the BCL including the suspensor, columella and quiescent center initials. Recently, 275 genes were found to be hypermethylated and downregulated in *ros1 dml2 dml3* triple mutant (*rdd*) seedlings undergoing tracheary element differentiation and were considered to be a subset of direct ROS1/DML2/DML3 targets (i.e. RDD targets) (Lin et al., 2020). We detected 50/275 RDD targets in ≥10% of nuclei in ≥1 embryonic cell cluster with enrichment scores ≥2 (Fig. 4B; Fig. S6). Sixteen of these RDD targets were highly enriched in the BCL. ROS1, DML2 and DML3 transcripts were increased specifically in the BCL between the one-cell and 32-cell stages (Zhou et al., 2020) (Fig. 4C). Consistently, most embryonic RDD target candidates were also increased in the BCL during these early embryonic stages (Fig. 4D). Although we could not detect morphological defects in *rdd* mutant embryos (Fig. S7), this may be due to redundancy with *DEMETER* (*DME*), which encodes a closely related DNA glycosylase family member (Choi et al., 2002; Gong et al., 2002). Consistent with this hypothesis, DME transcripts were enriched in the BCL and had increased levels in the BCL between the one-cell and 32-cell stages, similar to what we observed for ROS1, DML2 and DML3 (Fig. S7). Based on these results, we propose that DNA demethylases become activated in the BCL by the globular stage and catalyze the removal of methyl groups from a set of gene promoters to derepress their expression.

### Differential enrichment of TF binding motifs

**Fig. 5.**
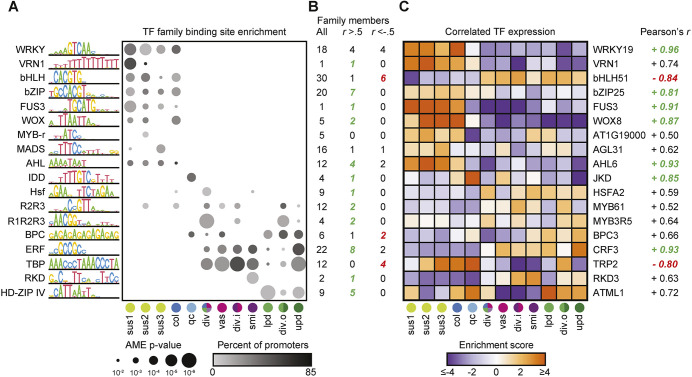
**Cluster-enriched TF binding motifs.** (A) Dot plot of TF families with DNA binding motifs significantly enriched in at least one cluster. Dot size shows the most significant enrichment (-log_10_ *P*-value, AME) of a motif in the family; dot color depicts the percentage of the top-250 ranked genes with a promoter containing the specified motif. (B) Number of TFs in each family that are detected in the globular atlas (left), have a Pearson's correlation between expression enrichment and motif enrichment across clusters greater than 0.5 (center), or less than −0.5 (right). (C) Heatmap of expression enrichment scores for the TF within each family with an expression enrichment that correlates most strongly to motif enrichment.

To gain insights into the transcriptional processes that help define these embryonic cell-specific transcriptomes, we tested whether any consensus DNA motifs from the CIS-BP database of TF binding experiments (Weirauch et al., 2014) were overrepresented in the promoters of the top-250 ranked genes of each cluster. We found that a total of 18 TF motif families were overrepresented in at least one cluster (Fig. 5A). Overrepresentation of a motif suggests that at least one of the TF family members influences the expression of the top-250 preferentially expressed genes of that cluster. Families of TFs that bind nearly identical motifs can be very large, making it difficult to determine which TF or TFs in a family could be interacting with a given motif. We sought to generate a collection of candidate genes most likely to be interacting with each significant motif in the embryo. We considered a TF a candidate if its binding motif exists in the CIS-BP database and was enriched, or if an enriched motif exists in the database for a TF in the same subfamily. We examined the correlations between TF family motif enrichments and the expression enrichments of individual TF candidates (Table S5; Fig. 5B) and highlighted the candidate that was most strongly positively or negatively correlated (Fig. 5C). For example, WRKY DNA-BINDING PROTEIN 2 (WRKY2) is a transcriptional activator in the BCL and was shown to directly activate *WOX8* and *WOX9* (Ueda et al., 2011). Consistent with this report, the most overrepresented motif in BCL clusters was the W-box bound by WRKY TFs, and this correlated well with the expression enrichment of *WRKY2* (Pearson's *r*=0.86; Table S5). In addition to *WRKY2*, the expression pattern of two other WRKY TFs (*WRKY28* and *WRKY19*) strongly correlated with enrichment of the WRKY motif (Pearson's *r*=0.94 and 0.96, respectively; Fig. 5B,C). The WOX family binding motif was similarly concentrated in BCL clusters, matching the observed expression pattern of *WOX8* (Fig. 5C) and to a lesser extent *WOX9*. The RNA encoding the B3 domain TF FUSCA3 (FUS3) is preferentially enriched in the BCL, and the RY motif bound by FUS3 is similarly enriched only in BCL clusters. Maintenance of quiescent center (QC) identity in roots requires *JACKDAW* (*JKD*), a member of the INDETERMINATE DOMAIN (IDD) subfamily of C2H2 zinc-finger TFs (Welch et al., 2007). The IDD motif is enriched exclusively in the QC initials, in which *JKD* is the second highest ranked gene behind *WOX5*. Class IV HOMEODOMAIN-LEUCINE ZIPPER (HD-ZIPs) include the L1 layer marker genes *MERISTEM LAYER 1* (*ATML1*) and *PROTODERMAL FACTOR 2* (*PDF2*), and their binding sites are overrepresented in the three protoderm clusters. The binding motifs of R1R2R3 Myb TFs, also known as mitosis-specific activator (MSA) elements, are enriched in the three clusters previously identified as actively dividing tissues (div, div.i, div.o), consistent with the role of R1R2R3 Myb TFs in positively regulating genes required for cytokinesis (Haga et al., 2007). Overall, the patterns of TF binding site enrichment are consistent with the literature on early embryo development, and the list of candidate TFs could serve as a valuable resource for future studies.

## DISCUSSION

We developed a method to generate high-quality transcriptomes from single embryonic nuclei without detectable contamination from surrounding seed tissues (Fig. 1). Individual nuclear transcriptomes were then grouped according to their cell type, which were validated using published datasets and RNA ISH (Fig. 2). This allowed us to construct a gene expression atlas of *Arabidopsis* embryos at the globular stage when the basic body plan is being established. Our results build upon foundational research examining the divergence of gene expression between the first two sporophytic cell lineages (Belmonte et al., 2013; Chen et al., 2021; Zhou et al., 2020) to help characterize how distinct gene expression programs, and corresponding cell types, are generated during early embryogenesis. Because evolutionary trajectories and transcripts encoding epigenetic factors or transcriptional regulators varied across early embryonic cell types, we surveyed these aspects to gain insights into how distinct gene expression programs are established in early embryos.

Consistent with a recent study, we found that genes preferentially expressed in suspensors tend to diverge more between species compared with those in embryo propers (Geist et al., 2019). Moreover, genes with enriched expression in columella initials were among the most rapidly evolving in the cell types we examined (Fig. 3C; Fig. S5). Conflict among siblings for maternal resources is thought to drive adaptive evolution of suspensors (Geist et al., 2019), which support the developing embryo proper and can serve as a conduit for maternally derived molecules (Nagl, 1990; Robert et al., 2018; Shi et al., 2019; Stadler et al., 2005; Yeung, 1980). Because columella initials are situated between the suspensor and embryo proper, they may help regulate communication between mothers and their offspring. In addition, DNA glycosylases required for demethylation of DNA are upregulated in the BCL of preglobular embryos, and our results are consistent with them catalyzing the removal of transcriptionally repressive methylation from gene promoters by the globular stage (Fig. 4). Interestingly, genes required for 24-nt siRNA biogenesis (e.g. *CLSY1*, *NRPD1A* and *DCL3*) were preferentially expressed in suspensors whereas transcripts encoding several AGO proteins that bind to small RNAs and mediate gene repression were enriched in embryo propers, especially shoot meristem initials from which the gametes are ultimately derived (Fig. 4A). Moreover, small RNAs can move between cells through plasmodesmata (Vatén et al., 2011), which also connect suspensors with embryo propers (Mansfield and Briarty, 1991). Similar to what has been proposed in other terminally differentiated cell-types in reproductive tissues (Calarco et al., 2012; Feng et al., 2013; Hsieh et al., 2009; Ibarra et al., 2012; Mosher and Melnyk, 2010; Slotkin et al., 2009), it is conceivable that suspensors generate large amounts of 24-nt siRNAs that flow into embryo propers and help silence and immobilize transposons to limit their mutagenic potential. Although beyond the scope of the current study, future cell-specific profiling of siRNAs and epigenetic marks in embryos should enable characterization of DNA demethylation and siRNA production in suspensors.

It is well-established that TFs drive pattern formation during animal embryogenesis, but relatively little is known about transcriptional regulation in plant embryos, partially due to redundancy among TF family members. By examining the relationships between TF expression levels and the enrichments/depletions of their corresponding binding motifs across embryonic cell types, we both verify existing models and provide testable hypotheses for how specific TFs influence cell-specific gene expression programs in early *Arabidopsis* embryos (Fig. 5; Table S5). For example, we observed characteristic patterns of TF binding motif enrichments and expression patterns in suspensors, quiescent center initials, sub-protoderm, protoderm and shoot meristem initials (Fig. 5). WRKY2 regulates suspensor development by transcriptionally activating *WOX8* and *WOX9*, which in turn are redundantly required for suspensor development (Breuninger et al., 2008; Ueda et al., 2011, 2017). Accordingly, suspensors were enriched for WRKY and WOX motifs, as well as motifs for FUS3 which has also been implicated in suspensor development (Lotan et al., 1998). Although VRN and AHL TFs do not have reported functions in suspensors, their binding motifs and expression of their corresponding family members (i.e. *VRN1*, *AHL1* and *AHL6*) were suspensor-enriched, which is consistent with transcriptional regulatory functions. TELOMERE BINDING PROTEIN (TBP) TFs have also not been implicated in suspensor development, and *TBP* expression and TBP binding motifs were enriched and depleted in suspensor-enriched gene promoters, respectively. TBPs can recruit polycomb group complexes (PcGs) to target loci and help repress their expression (Zhou et al., 2016, 2018). Similarly, BASIC PENTACYSTEINE (BPC) TFs can also recruit PcGs to target loci (Hecker et al., 2015; Xiao et al., 2017), and expression of specific BPC family members (e.g. *BPC5/7*) was enriched in suspensors, and BPC binding motifs were depleted from the promoters of top-250 ranked suspensor genes (Table S5). Future experiments are required to test whether TBP/BPC-mediated recruitment of PcGs and resulting epigenetic silencing is required for suspensor development.

The QC initials are derived from the uppermost derivative of the BCL and, unlike suspensors, contribute to post-embryonic tissues. IDD TF binding motifs were specifically enriched in the QC initials and expression of the IDD family member, JKD, which is required for QC identity in roots (Welch et al., 2007), was highly enriched in QC initials but not suspensors (Fig. 5). This implies that the superimposition of JKD on the BCL TF combinations could promote QC initial identity in early embryos. In contrast to the BCL, the sub-protoderm (i.e. inner cells of the embryo proper) is enriched in motifs including those for BPC, TBP and ERF TFs (Fig. 5). Among several other ERF family members preferentially expressed in the embryo proper, DRN functions upstream of auxin and binds GCC motifs to promote meristem identity (Chandler et al., 2007; Eklund et al., 2011; Iwase et al., 2017; Kirch et al., 2003). The protoderm already expresses genes characteristic of specific processes inherent to the outermost layer during early embryogenesis (Fig. 3A) and is enriched for HD-ZIP IV TF motifs (Fig. 5A). Accordingly, transcripts encoding ATML1 and PDF2 family members were enriched in the protoderm and are required for its specification (Abe et al., 2003; Ogawa et al., 2015). Another cell-specific enrichment of cis-regulatory motifs was observed for RKD TFs in the shoot meristem initials. RKD genes tend to be expressed in reproductive tissues of land plants (Jeong et al., 2011; Koi et al., 2016; Kőszegi et al., 2011; Waki et al., 2011) and their overexpression is sufficient to induce expression of undifferentiated cell types (Kőszegi et al., 2011; Waki et al., 2011). Therefore, the enrichment of RKD motifs, as well as the preferential expression of *RKD3/5* family members, in the shoot meristem initials make *RKD3/5* good candidates for future investigation into the establishment of shoot meristem initial gene expression programs.

In addition to providing an early embryonic gene expression atlas, the presented workflow may help guide snRNA-seq experiments on embryos and other plant tissues that are difficult to access. The future application of similar techniques across embryonic stages in *Arabidopsis* and other species should contribute to a deeper understanding of how gene expression programs are dynamically established during plant embryogenesis. Moreover, integrating snRNA-seq data with other single-cell genomic technologies such as single-cell ATAC-seq (Buenrostro et al., 2015; Cusanovich et al., 2015) may allow further characterization of gene regulatory mechanisms operating in plant embryos. We expect that, together with more focused studies, these genome-wide datasets will accelerate our understanding of the molecular basis of pattern formation in plant embryos.

## MATERIALS AND METHODS

### Plant materials, growth conditions and microscopy

*Arabidopsis thaliana* accession Columbia (Col-0) plants containing pWOX2::H2B-GFP, pWOX2::tdTomato-RCI2b (pWOX2::NLS-GFP) (Gooh et al., 2015) or no transgenes were grown at 20-22°C and 16 h light/8 h dark cycles under incandescent lights (130-150 µmol/m^2^/s) in a climate-controlled growth chamber. The *rdd* triple mutants were composed of *ros1-3*, *dml2-1* and *dml3-1* (Penterman et al., 2007) and Nomarski microscopy was carried out as previously described (Plotnikova et al., 2019).

### Nuclei isolation and FANS

Developing seeds containing globular embryos from the transgenic pWOX2::H2B-GFP, pWOX2::tdTomato-RCI2b lines and wild-type Col-0 were isolated before sorting. For each set, developing seeds were isolated with tungsten needles under a stereomicroscope from 20 self-pollinated siliques at stage 17 (Smyth et al., 1990), corresponding to 72 h after pollination when most embryos are at the early/mid-globular stage under the growth conditions used. Developing seeds were isolated at the same time of day to minimize variations caused by circadian rhythms and immediately transferred to 600 μl cooled fixative buffer consisting of 1× Galbraith's buffer [20 mM MOPS (pH 7.0), 30 mM sodium citrate, 1% Triton X-100, 45 mM MgCl_2_] and 500 µM dithiobis(succinimidyl propionate) (DSP; Thermo Fisher Scientific). All buffers used in the nuclei isolation and sorting contained 0.4 U/ml RNAse inhibitor murine (New England Biolabs). Cross-linked samples were incubated with 800 μl quenching buffer [1 M Tris-HCl (pH 7.0), 30 mM sodium citrate, 1% Triton X-100 and 45 mM MgCl_2_] at room temperature for 15 min with gentle shaking. The quenched samples were washed twice with 600 μl HG-GB (1× Galbrath's buffer and 1 M hexylene glycerol; Sigma-Aldrich). The seeds were then gently homogenized with micro-pestles in 1.5 ml microtubes with 200 μl HG-GB. Micro-pestles were rinsed with 400 μl HG-GB, and the homogenized samples were gently pipetted ten times before incubating at 4°C for 15 min to maximize nuclei release. The partially homogenized samples were then filtered with 30 μm filters and collected in 2 ml microtubes. Another 600 μl HG-GB were added to the 1.5 ml microtube, and filtered and collected through the same 30 μm filter and 2 ml microtube, respectively, to maximize nuclei recovery. The filtered samples were then centrifuged at 1000 ***g*** at 4°C for 10 min. The supernatant was carefully removed without disturbing the grayish pellet of nuclei. A fresh aliquot of 1 ml HG-GB and 1 μl of 10 mg/ml DAPI was added into microtubes and the pellet was gently re-suspended. Samples were then washed five times, including a 10-min centrifugation at 1000 ***g*** at 4°C and replacement of supernatant with fresh aliquots of 1 ml 1× Galbrath's buffer. The washed nuclei were then re-suspended in 800 μl 1× Galbraith's buffer for sorting.

The isolated nuclei were sorted with a BD FACSAria™ III Cell Sorter (BD Biosciences) with a 70 μm nozzle. The scatter gates were adjusted accordingly with Col-0 nuclei. DAPI signals were activated by a 375 nm laser and collected with a 450/40 nm filter. GFP signals were activated by a 488 nm laser and collected with a 530/30 nm filter. To maximize purity, only the droplets containing a DAPI signal within the two peak regions representing 2 constant (2C) and 4 constant (4C) nuclei (Fig. S1A,C) were considered for GFP gating. For GFP gating, a region with low auto-fluorescence and high GFP signal was selected (Fig. S1B,D), which had less than three events in Col-0 samples and on average ≥200 events for pWOX2::NLS-GFP samples. Each nucleus passing both DAPI and GFP gating was collected with single-cell settings in 4 μl of cell lysis buffer (Picelli et al., 2014a) supplemented with 25 mM DTT in single wells of 96-well plates.

### snRNA-seq

Smart-seq2 libraries were prepared following the published SmartSeq2 single-cell protocol (Picelli et al., 2014a,b) with an additional 30-min 37°C incubation before reverse transcription. Libraries were sequenced on an Illumina Hi-Seq 2500 in 50-base single end mode. Sequencing reads from each sample were preprocessed by trimming adapters using cutadapt v2.6 (Martin, 2011) in two steps. First, Nextera adapters (5′-CTGTCTCTTATACACATCTCCGAGCCCACGAGAC-3′) were trimmed from the 3′ end of reads, followed by trimming of template-switching oligos (TSO; 5′-AAGCAGTGGTATCAACGCAGAGTACATGGG-3′) and oligo-dT adapters (5′-AAGCAGTGGTATCAACGCAGAGTACTTTTTTTTTTTTTTTTTTTTTTTTTTTTTT-3′) from the 5′ and 3′ ends of reads, respectively. A Kallisto index was built from a combined FASTA file of all transcript models in EnsemblPlants TAIR10 v40 (ftp://ftp.ensemblgenomes.org/pub/plants/release-40/gff3/arabidopsis_thaliana/Arabidopsis_thaliana.TAIR10.40.gff3.gz), 96 ERCC spike-in transcripts and sGFP. Each trimmed sample FASTQ file was pseudoaligned to this index using the command ‘kallisto quant’ with the options ‘--single --fragment-length 200 --sd 100’ to produce a table of transcripts per million (TPM) for each sample.

### Quality control and census count conversion

The TPM table, cell data and gene data were imported into Monocle3 (Cao et al., 2019). Libraries with less than either 100,000 aligned reads or 1000 detected genes were considered as low quality and excluded from subsequent analyses. The TPM values were then converted to census counts with the census conversion algorithm (Qiu et al., 2017; Trapnell et al., 2014). The census counts were used as gene expression levels in the subsequent analyses.

### Maternal contamination removal and tissue enrichment tests

Gene expression values were used to perform tissue enrichment tests with default settings as described (Schon and Nodine, 2017). The census count expression and metadata of snRNA-seq libraries from eight plates (Table S1) were constructed as a cell data set (CDS) in Monocle3 with R version 3.6.3. The quality control was carried out according to Monocle3 guidelines. Genes passing the Monocle3 function detect_genes(CDS, min_expr=0.1) and expressed in at least three nuclei were considered in subsequent analyses. The above quality control steps resulted in a CDS with 534 nuclei and 24,591 genes. An unsupervised UMAP (McInnes et al., 2018 preprint) dimension reduction and clustering performed on this CDS resulted in 20 clusters. Two of the clusters (Clusters 12 and 13 in Fig. S2) were dominated by nuclei that resembled the seed coat reference according to tissue enrichment tests, and therefore corresponding nuclei were excluded from subsequent analyses. After contamination removal, a CDS containing 486 globular embryonic nuclei and 23,959 detectable genes was then used for subsequent cell type score calculation and clustering.

### Calculation of cell type scores for globular nuclei and clustering

A set of 174 embryonic marker genes based on either RNA ISH or transcriptional/translational fusions to fluorescent or beta-glucuronidase (GUS) reporters were collected from the literature (Table S2). Expression levels were recorded as strongly expressed (s), weakly expressed (w), not expressed (n) or non-informative (NA) for each of nine cell types: upd, lpd, smi, upper inner periphery (uip), vas, ground tissue initials (grd), qc, col and sus. The corresponding 174×9 matrix was intersected with expressed genes in our globular snRNA-seq libraries, which had at least one census count in at least seven nuclei. The resulting 135 expressed marker genes served as the reference for cell type-score calculations, with 56, 52, 38, 43, 62, 43, 56, 51 and 29 positive markers (i.e. strongly or weakly expressed) and 79, 83, 97, 92, 73, 92, 79, 84 and 98 negative markers (i.e. not expressed) for upd, lpd, smi, uip, vas, grd, qc, col and sus, respectively. We used two-tailed hypergeometric tests assuming that a nucleus expressing positive and negative markers of a cell type was more or less likely to be from that cell type, respectively. The resulting *P*-values were -log_10_-transformed to compute cell type scores. The 486×9 matrix of cell type scores was then used for dimension reduction and clustering. Cluster identities were predicted based on the cell type labels within each cluster.

### Validation of cluster identities

The mean expression values of all nuclei within each cluster were used to perform tissue enrichment tests as previously described (Schon and Nodine, 2017) and to calculate Spearman's correlation coefficients with published globular stage embryo proper (32E) and suspensor (32S) samples (Zhou et al., 2020). The expression levels of selected markers and three recently reported genes [*PEAR1* (*AT2G37590*), *DOF6* (*AT3G45610*) and *GATA20* (*AT2G18380*)] (Smit et al., 2020) not included in our reference marker table for tissue score calculation were plotted with the Monocle3 ‘plot_genes_by_group()’ function.

We selected 33 RNA ISH candidates without previously reported embryonic expression patterns according to their expression patterns and probe specificity (Table S6). RNA *in situ* probes were generated from synthesized double-stranded DNA (gBlocks Gene Fragments; Integrative DNA Technologies) and applied as previously described (Nodine et al., 2007). For each probe, 21-122 globular stage embryos (i.e. biological replicates) were examined from two to eight microscope slides (i.e. technical replicates) for a total of 1420 embryos from 112 slides (Fig. S4). To minimize potential bias, all *in situ* images were examined and classified by someone that did not perform the experiments and did not know the identities of the samples.

### Ranked gene enrichment

For each cluster of nuclei, a ranked gene enrichment strategy was defined as follows: let *G* be the set of ‘expressed’ genes, defined as all nuclear-encoded and RNA Polymerase II-transcribed genes with ≥1 RNA-seq read count in ≥10% of nuclei in ≥1 cluster. For each gene *i* in each nucleus *j*, 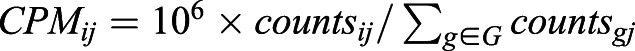. Let *C* be a set of nuclei in a cluster and |*C*| the number of nuclei in cluster *C*. Mean CPM of gene *i* in cluster *C* is defined as 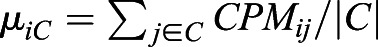. Proportion detected *p* is defined for each gene *i* in each cluster *C* as the number of nuclei in which gene *i* was detected: 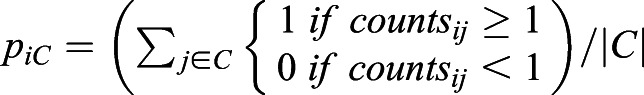. Using one cluster *C* as an ingroup and all other clusters as outgroup *O*, a mean CPM log_2_ fold change of each gene *i* is calculated as 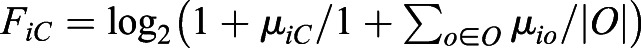, and a mean proportion difference 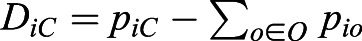. Both sets *F_C_* and *D_C_* were centered and mean-scaled so that 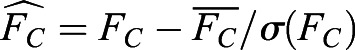, and 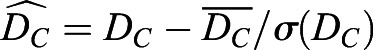, where 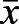 is the mean and *σ*(*x*) the standard deviation. Enrichment magnitude *E_iC_* of gene *i* in cluster *C* is the combined deviation from the mean of *F_C_* and *D_C_*:
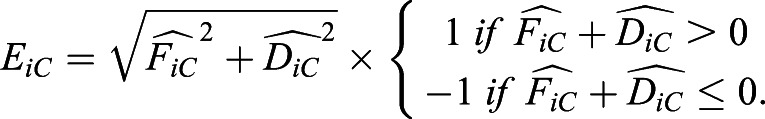
In each cluster, genes were ranked from highest to lowest enrichment magnitude and the first 250 genes and last 250 genes were considered ‘top-ranked genes’ and ‘bottom-ranked genes’, respectively (Table S3).

### Gene ontology analyses

The IDs of the top-250 ranked genes for each cluster were submitted to TAIR GO Term enrichment (https://www.arabidopsis.org/tools/go_term_enrichment.jsp) using the PANTHER classification system (Mi et al., 2021) to compute false discovery rates with Fisher's exact tests. All enriched terms are presented in Table S4. The five most significant GO terms not related to ribosomes are highlighted in Fig. 3.

### TF binding site analyses

Promoters for all genes were defined as the region 500 bp upstream to 100 bp downstream of the most common 5′ end in nanoPARE datasets of globular-stage embryos (Plotnikova et al., 2019). For genes without nanoPARE signal, the most upstream 5′ end annotated in TAIR10 v.46 was used. TF binding motifs for *Arabidopsis thaliana* were downloaded from CIS-BP (http://cisbp.ccbr.utoronto.ca) (Weirauch et al., 2014). All directly determined motifs were tested for statistical overrepresentation using Analysis of Motif Enrichment (AME; http://meme-suite.org/doc/ame.html) (McLeay and Bailey, 2010) in each cluster by comparing the top-250 ranked gene promoters against a background set of the bottom-250 ranked gene promoters with default parameters. Motifs that were significantly enriched in at least one cluster were collapsed into motif families. The cluster-specific expression of all genes with a significantly enriched motif were tested for correlation with the cluster-specific pattern of motif family enrichment, as well as all genes not represented in the CIS-BP database, but in the same TF subfamily as a gene with a significant motif.

## Supplementary Material

Supplementary information

Reviewer comments
