# Supplementary material for: Gene expression variation in Arabidopsis embryos at single-nucleus resolution
Source: Development. 2021 Jul 6;148(13):dev199589. doi: 10.1242/dev.199589 (PMC8276985; doi:10.1242/dev.199589)
Supplement: Reviewer comments [file develop-148-199589_review_history.pdf]

Gene expression variation in *Arabidopsis* embryos at single-nucleus resolution

Ping Kao, Michael A Schon, Magdalena Mosiolek, Balaji Enugutti and Michael D. Nodine
DOI: 10.1242/dev.199589

Editor: Yka Helariutta

Review timeline
Original submission: 5 March 2021
Editorial decision: 16 April 2021
First revision received: 12 May 2021
Accepted: 24 May 2021

Original submission

First decision letter

MS ID#: DEVELOP/2021/199589

MS TITLE: Gene Expression Variation in Arabidopsis Embryos at Single-Nucleus Resolution

AUTHORS: Ping Kao, Michael A Schon, Magdalena Mosiolek, and Michael D Nodine

I have now received all the referees' reports on the above manuscript, and have reached a decision. The referees' comments are appended below, or you can access them online: please go to BenchPress and click on the 'Manuscripts with Decisions' queue in the Author Area.

As you will see, the referees express considerable interest in your work, but have some significant criticisms and recommend a substantial revision of your manuscript before we can consider publication. If you are able to revise the manuscript along the lines suggested, which may involve further experiments, I will be happy receive a revised version of the manuscript. Your revised paper will be re-reviewed by one or more of the original referees, and acceptance of your manuscript will depend on your addressing satisfactorily the reviewers' major concerns. Please also note that Development will normally permit only one round of major revision.

We are aware that you may be experiencing disruption to the normal running of your lab that make experimental revisions challenging. If it would be helpful, we encourage you to contact us to discuss your revision in greater detail. Please send us a point-by-point response indicating where you are able to address concerns raised (either experimentally or by changes to the text) and where you will not be able to do so within the normal timeframe of a revision. We will then provide further guidance. Please also note that we are happy to extend revision timeframes as necessary.

Please attend to all of the reviewers' comments and ensure that you clearly highlight all changes made in the revised manuscript. Please avoid using 'Tracked changes' in Word files as these are lost in PDF conversion. I should be grateful if you would also provide a point-by-point response detailing how you have dealt with the points raised by the reviewers in the 'Response to Reviewers' box. If you do not agree with any of their criticisms or suggestions please explain clearly why this is so.

© 2021. Published by The Company of Biologists under the terms of the Creative Commons Attribution License (https://creativecommons.org/licenses/by/4.0/).

Reviewer 1

Advance summary and potential significance to field

The reviewed manuscript "Gene Expression Variation in Arabidopsis Embryos at Single-Nucleus Resolution" by Kao et al, presents the first report (to my knowledge) of the use of single nuclei RNAseq technology to accurately map gene expression in a tissue-specific manner in plant embryos. This is an advancement from previous RNAseq experiments from either isolated embryo or laser-dissected embryos (or seed tissues/parts). Even if the study is focused on globular embryos, due to the amount of RNAseq libraries used the choice of globular embryos over the other stages is relevant from a developmental point of view.
This work is a significant improvement from what is already published. As noted by the authors (and demonstrated in the study), it avoids contamination from non-zygotic embryonic tissues. This issue has been a problem from the many other studies, which could have led to misinterpretation of functional analysis from a specific set of genes. From a technical point of view, this is a challenging methodology applied to a tricky tissue (globular embryos). Therefore, this study is highly significant for researchers in the field and beyond.
To mention also, that data fully support all drawn conclusions.

Comments for the author

The manuscript is complete. The text is coherent and properly written. I would have only minor comments related to the text.

For example, I would add single nuclei RNAseq in the keyword list to highlight the technological advances of the presented work.

In line 198, the authors mentioned the discrepancy between the RNAseq data and the ISH data that it may be caused by post-transcriptional regulation. True. But what about the bias about the methodology looking only at nuclear mRNA molecules and losing all information about the cytoplasmic mRNA? How much could it be relevant to explain the minor differences?

Line 80, I would replace "the most fundamental plant tissues" with more descriptive wording. What are these fundamental plant tissues?

Concerning panel 2G, it actually took me a while to notice it (I thought it was missing). It would be worth making it more visible somehow. The legend also mentions some asterisks that are missing from the figure.

And legends of Fig S5 and S6 are referring to 1G instead of 2G.

Also, I am not about the policy of Development in this aspect, but are all the figure panels referred to in the text? I think this concerns Fig 5 and Table S3.

And the quality of some of the pictures presenting ISH is not great, notable in Supp figure S3. It is sometimes difficult to see any signal. However, I appreciated the quantitative analysis in the supplemental figure S4.

Reviewer 2

Advance summary and potential significance to field

Kao et al report RNA-sequencing of single nuclei prepared form globular-stage Arabidopsis embryos. Nuclei were purified by fluorescence-activated sorting from whole seed using an embryo-specific marker; prior to processing, seeds were treated with the reversible fixative DSP, recently reported to protect RNA integrity in single cell or single nucleus techniques. The authors employed stringent quality control to eliminate libraries with low read count and libraries that were likely derived from contaminating seed coat material. The remaining 486 libraries had an average read count of about

© 2021. Published by The Company of Biologists under the terms of the Creative Commons Attribution License (https://creativecommons.org/licenses/by/4.0/).

½ million mapping to more than 2500 genes – these numbers compare favorably to the very few reports of similar experiments with plant nuclei.

It was not possible to resolve the nine cell types expected to be represented in globular embryos by unsupervised clustering of the overall expression data. Instead, clusters were generated with the aid of 135 marker genes with known expression patterns collected form the literature, by what seems to be a clever probabilistic procedure. The resulting clusters seem realistic (although see below).

Further analysis led to the discovery of many novel cell type-specific transcripts. The expression pattern of these transcripts in globular embryos was experimentally verified by the best method available, in-situ hybridization (a substantial effort). In a second, more speculative. part of the manuscript, preferential expression of epigenetic regulators in the two main domains (basal/suspensor and apical/proembryo) is linked to a potential division of labor in the production of small RNAs targeting transposons as well as to the overall differentiation state; and prominently expressed transcription factors are linked to the refinement of spatial domains by correlating the expression of potential target genes with the presence of predicted binding sites in their promoter regions.

Overall, we believe this is a valuable and important contribution. The technical approach documented here could pave the way for a comprehensive characterization of embryonic transcriptional networks at a cellular resolution. As is, the data set arguably provides the most detailed characterization of global transcription in globular plant embryos to date and will be very useful to the community (the supplemental tables are well-
organized in this respect).

Comments for the author

Suggestions for revision:
The authors should comment on why the overall expression data did not result in informative clusters. Was it likely due to a technical problem (noise, sparse data), or did individual nuclei actually show mixed, not clearly specified, or transient fates? The matrix of cell type- should be added to the supplemental material.

The last two sections of the result section, discussing epigenetic regulators and TF targets, contain little validation. Maybe some of the conclusions should be phrased more cautiously ("…")?

Specifically:
306: "ros1 dml2 dml3 triple mutant seedlings". Again, the trends seen with targets of RDD are interesting, but the significance of this observation is not quite clear. If triple mutants are available, the authors could assess their suspensor development – any defects would imply functional relevance and strengthen their case. (If no defects can be found, what would remain of the argument?).
318: "Differential enrichment of transcription factor binding motifs." We are not entirely sure how to interpret these observations. Many of the TF families are quite large and contain members that have diverse functions and expression patterns. For example, most WOX genes are not expressed in the suspensor and have been shown to function in proembryo patterning. Given this, what does it mean that enrichment of WOX binding motifs is observed in genes preferentially expressed in the basal cell lineage? Similarly, the bHLH genes TMO5 and LHW are certainly important for vascular tissue specification – so what do we make of the finding that enrichment of bHLH motifs is observed in genes preferentially expressed in the suspensor?

Details:
168 and 212: "Cluster 9 had mixed cell type assignments" & "clusters 1, 5 and 9 represent dividing subprotoderm". Does this mean that dividing cells in the embryo proper and suspensors tend to lose their cell-fate markers?
188: "AT1G044880" – is this "AT1G04880", as in Fig. 2F?
214: "Altogether, our in silico and in situ validations indicated that. we can assign […] libraries to the major cell types." It would be good if the authors could clarify minor discrepancies between results and expectations. Three clusters corresponding to "suspensor" were found – why, are there

© 2021. Published by The Company of Biologists under the terms of the Creative Commons Attribution License (https://creativecommons.org/licenses/by/4.0/).

really three different cell types in the suspensor? No clusters corresponding to “upper inner periphery” and “upper protoderm” were found – why, not enough nuclei?
224: “13,893 transcripts detected in ≥10% of nuclei in ≥1 cluster”. Is it possible to be in ≥10% of nuclei in <1 cluster? 287 & 303: expression of CLSY1, NRPD1A, DCL3, ROS1, DML2, DML3. It is interesting finding that these genes are enriched in the basal cell lineage, but no validation is presented. ISH would be ideal but maybe too much of an effort; could published profiles be consulted? 287 and elsewhere: “terminally differentiated suspensor”. We are not sure it is fair to say that the suspensor cells of globular embryos are terminally differentiated. For example, the scatter plots do not seem to suggest that the nuclei were polyploid yet.

Figure 3A – enriched GO terms. Microsporocyte differentiation is an unexpected category. Any explanation?

Fig. S2: “C” in the legend seems to correspond to “D” in the figure?
The recent paper by Farmer et al. should be added to the references (doi: 10.1016/j.molp.2021.01.001).

Reviewer 3

Advance summary and potential significance to field

In this manuscript by Kao et al., the author present what I believe is the first data on single cell sequencing of Arabidopsis globular stage embryos. The authors use a pWOX2::H2B-GFP transgenic line in order to isolate nuclei specifically from the developing embryo and not the surrounding maternal tissue and endosperm. The authors then isolated RNA from these nuclei and perform several quality control measure and ultimately generated 486 single cell libraries. Library sequences were binned according to the co-expression of known markers for the embryo and were able to make informed predictions as to where multiple new genes would be expressed. The authors show a nice correlation between their cell type predictions by presenting high quality in situ hybridization data in figure 2. They were clearly able to distinguish between RNAs found in the suspensor vs the embryo proper as well as subsets of cell types such as the protoderm. They then present an analysis of epigenetic regulators and their preferential accumulation pattern which is interesting. A final analysis of transcription factor binding motifs is also presented.

Overall, the data appears of high quality and the Nodine group is known for their work on early embryonic gene expression and have shown they have the technical skills required to perform these types of analyses that are embryo specific and not contaminated with other tissue. The datasets presented will be a valuable resource for those working on this stage of embryogenesis, although overall the paper feels a bit thin in comparison to recently published single cell RNA Seq papers that are being published at a rapid pace. The confirmation of cell type expression is nice, but really no further functional data is presented.

Comments for the author

My main concern is the final analysis in the section entitled “Differential enrichment of transcription factor binding motifs”. I am not sure this really adds much to the paper and seems highly speculative, especially the last section on the co-occurance of ERF binding sites and teloboxes. This last example could be deleted without affecting the take homes of the manuscript.

© 2021. Published by The Company of Biologists under the terms of the Creative Commons Attribution License (https://creativecommons.org/licenses/by/4.0/).

First revision

Author response to reviewers' comments

Thank you for handling our manuscript and to the Reviewers for their time and constructive comments. We are pleased with the favorable reviewer comments and have addressed each of the questions raised in the revised manuscript as detailed below. Please find the reviewer comments in black and our responses inserted in blue. In the accompanying files, we have provided a word doc with highlighted changes, a submission checklist, as well as manuscript text, figures and supplementary materials formatted according to Development's guidelines. We believe that we have fully addressed each of the referees' comments and hope that you will now find it suitable for publication in Development.

Reviewer 1 Advance summary and potential significance to field...
The reviewed manuscript "Gene Expression Variation in Arabidopsis Embryos at Single-Nucleus Resolution" by Kao et al, presents the first report (to my knowledge) of the use of single nuclei RNAseq technology to accurately map gene expression in a tissue-specific manner in plant embryos. This is an advancement from previous RNAseq experiments from either isolated embryo or laser-dissected embryos (or seed tissues/parts). Even if the study is focused on globular embryos, due to the amount of RNAseq libraries used, the choice of globular embryos over the other stages is relevant from a developmental point of view.
This work is a significant improvement from what is already published. As noted by the authors (and demonstrated in the study), it avoids contamination from non-zygotic embryonic tissues. This issue has been a problem from the many other studies, which could have led to misinterpretation of functional analysis from a specific set of genes. From a technical point of view, this is a challenging methodology applied to a tricky tissue (globular embryos). Therefore, this study is highly significant for researchers in the field and beyond. To mention also, that data fully support all drawn conclusions.

We are glad that you appreciate our work and have addressed each of your comments as described below.

Reviewer 1 Comments for the author...

The manuscript is complete. The text is coherent and properly written. I would have only minor comments related to the text.
For example, I would add single nuclei RNAseq in the keyword list to highlight the technological advances of the presented work.

We have added "single-nucleus RNA-seq" to the keywords in the revised manuscript to highlight the corresponding technological advances.

In line 198, the authors mentioned the discrepancy between the RNAseq data and the ISH data that it may be caused by post-transcriptional regulation. True. But what about the bias about the methodology looking only at nuclear mRNA molecules and losing all information about the cytoplasmic mRNA? How much could it be relevant to explain the minor differences?

Because our method captured nuclear polyadenylated RNAs and polyadenylation is coupled to nuclear export, the nuclear mRNAs we profiled were likely rapidly exported to the cytoplasm. However, we cannot completely rule out that differences in the RNA-seq and ISH data are due to differences in nuclear vs. cytoplasmic mRNA populations not related to post-transcriptional regulation and have added a clarifying statement on lines 136-139 in the revised manuscript.

Line 80, I would replace "the most fundamental plant tissues" with more descriptive wording. What are these fundamental plant tissues?

We agree and have used more descriptive wording regarding these fundamental plant tissues on lines 82-83 in the revised manuscript.

© 2021. Published by The Company of Biologists under the terms of the Creative Commons Attribution License (https://creativecommons.org/licenses/by/4.0/).

Concerning panel 2G, it actually took me a while to notice it (I thought it was missing). It would be worth making it more visible somehow. The legend also mentions some asterisks that are missing from the figure.

This is a good point. To make panel 2G more visible we have outlined it with a box in the revised manuscript. We had mistakenly included the mention of asterisks from a previous version of the manuscript and have removed them in the revised one.

And legends of Fig S5 and S6 are referring to 1G instead of 2G.

Thank you for spotting these errors. We have corrected them in the revised supplementary information document.

Also, I am not about the policy of Development in this aspect, but are all the figure panels referred to in the text? I think this concerns Fig 5 and Table S3.

We included citations for Table S3, Fig. 5B and Fig. 5C in the revised manuscript.

And the quality of some of the pictures presenting ISH is not great, notable in Supp figure S3. It is sometimes difficult to see any signal. However, I appreciated the quantitative analysis in the supplemental figure S4.

Although we were quite pleased with the quality of ISH images presented in Fig. 2, we agree that not all ISH images in Fig. S3 were great, which was primarily due to low signal. However, these were the representative images. RNA in situs on sections of early Arabidopsis embryos is a difficult technique, which is why we examined many embryos (1,420 from 112 slides), and categorized the resulting patterns independent of knowing the probe identities (Fig. S4). We are glad that you, as well as Reviewer #2, appreciate the corresponding quantitative analysis in Fig. S4 and this unbiased assessment gave us confidence in the conclusions drawn.

Reviewer 2 Advance summary and potential significance to field...
Kao et al report RNA-sequencing of single nuclei prepared form globular-stage Arabidopsis embryos. Nuclei were purified by fluorescence-activated sorting from whole seed using an embryo-specific marker; prior to processing, seeds were treated with the reversible fixative DSP, recently reported to protect RNA integrity in single cell or single nucleus techniques. The authors employed stringent quality control to eliminate libraries with low read count and libraries that were likely derived from contaminating seed coat material. The remaining 486 libraries had an average read count of about ½ million mapping to more than 2500 genes – these numbers compare favorably to the very few reports of similar experiments with plant nuclei.

It was not possible to resolve the nine cell types expected to be represented in globular embryos by unsupervised clustering of the overall expression data. Instead, clusters were generated with the aid of 135 marker genes with known expression patterns collected form the literature, by what seems to be a clever, probabilistic procedure. The resulting clusters seem realistic (although see below).

Further analysis led to the discovery of many novel cell type-specific transcripts. The expression pattern of these transcripts in globular embryos was experimentally verified by the best method available, in-situ hybridization (a substantial effort). In a second, more speculative. part of the manuscript, preferential expression of epigenetic regulators in the two main domains (basal/suspensor and apical/proembryo) is linked to a potential division of labor in the production of small RNAs targeting transposons as well as to the overall differentiation state; and prominently expressed transcription factors are linked to the refinement of spatial domains by correlating the expression of potential target genes with the presence of predicted binding sites in their promoter regions.

Overall, we believe this is a valuable and important contribution. The technical approach documented here could pave the way for a comprehensive characterization of embryonic transcriptional networks at a cellular resolution. As is, the data set arguably provides the most

© 2021. Published by The Company of Biologists under the terms of the Creative Commons Attribution License (https://creativecommons.org/licenses/by/4.0/).

detailed characterization of global transcription in globular plant embryos to date and will be very useful to the community (the supplemental tables are well-organized in this respect).

Thank you very much for the positive comments. We are happy that you value our efforts and we have tried our best to address each of your comments below.
Reviewer 2 Comments for the author...

Suggestions for revision:

The authors should comment on why the overall expression data did not result in informative clusters. Was it likely due to a technical problem (noise, sparse data), or did individual nuclei actually show mixed, not clearly specified, or transient fates? The matrix of cell type- should be added to the supplemental material.

This is an excellent point. Although we do not know why unsupervised UMAP clustering could not resolve individual embryonic cell types, we suspect that it is either due to the relatively low number of snRNA-seq libraries included in the clustering (n = 486) or because the embryonic cell fates are transient in these emerging cell types. We have commented on this on lines 136-139 in the revised manuscript. The cell type score matrix and the matrix of markers across known embryonic cell types are shown in Table S1 and Table S2, respectively.

The last two sections of the result section, discussing epigenetic regulators and TF targets, contain little validation. Maybe some of the conclusions should be phrased more cautiously ("…")?

Specifically:

Thank you for the input. Reviewer #3 also thought aspects of the TF target section were a bit too speculative and we have edited the corresponding figure (Fig. 5) and text.

306: "ros1 dml2 dml3 triple mutant seedlings". Again, the trends seen with targets of RDD are interesting, but the significance of this observation is not quite clear. If triple mutants are available, the authors could assess their suspensor development – any defects would imply functional relevance and strengthen their case. (If no defects can be found, what would remain of the argument?).

Triple ros1 dml2 dml3 (rdd) mutants are available and we have examined rdd embryos. Although we did not observe phenotypes in rdd embryos, this may be due to genetic redundancy with DEMETER (DME), which encodes a closely related DNA glycosylase family member required for endosperm development and pollen tube growth. Consistent with this possibility, and similar to ROS1, DML2 and DML3, DME expression was also enriched in the basal cell lineage (BCL) and increased between the 1-cell and 32-cell stages. We have included these new results in Fig. S7 and corresponding text of the revised manuscript (lines 319-324).

318: "Differential enrichment of transcription factor binding motifs." We are not entirely sure how to interpret these observations. Many of the TF families are quite large and contain members that have diverse functions and expression patterns. For example, most WOX genes are not expressed in the suspensor and have been shown to function in proembryo patterning. Given this, what does it mean that enrichment of WOX binding motifs is observed in genes preferentially expressed in the basal cell lineage? Similarly, the bHLH genes TMO5 and LHW are certainly important for vascular tissue specification – so what do we make of the finding that enrichment of bHLH motifs is observed in genes preferentially expressed in the suspensor?

We agree that it can be difficult to interpret all the results shown in Fig. 5 and have included text in the revised manuscript (lines 332-343) to help clarify the limits of this analysis and give more detail on why a gene might be included or excluded from the analysis. It is true that we do not know exactly which TF family members activate or repress the preferentially expressed genes for each cluster (i.e. top-250 ranked genes) in which their binding motifs were significantly enriched. Therefore, we provided a list of candidates in Table S5 for future studies and highlighted the family member whose expression enrichment correlates most strongly to motif enrichment in Fig. 5C. In fact, the results from our analysis of WRKY, FUS3, WOX, IDD and HD-ZIP IV families support

© 2021. Published by The Company of Biologists under the terms of the Creative Commons Attribution License (https://creativecommons.org/licenses/by/4.0/).

our approach based on the literature. For example, the enrichment of WOX binding motifs observed in genes preferentially expressed in the basal cell lineage suggests that at least one WOX family member contributes to the preferential expression of these top-250 genes in the basal cell lineage. We suggest that WOX8/9 are the most likely candidates in this case based on their expression correlation (Table S5), which is consistent with previous reports that WOX8/9 are expressed and redundantly required for suspensor development (Breuninger et al., 2008; Haecker et al., 2004; Ueda et al., 2011).

Because the bHLH family is composed of the most members out of all the families (n = 161) that we found significant motif enrichments for, it is perhaps one of the most difficult ones to predict the precise family members involved. Only 57 bHLH family members have entries in the CIS-BP database, and 43 of these were significant. We did not include LHW in our analysis because it is not present in the CIS-BP database and the most closely related gene (AT1G64625) in the database had a motif that did not match the enriched bHLH motif. However, we observed several bHLH family members that were either enriched (e.g. LRL2, SPT) or depleted (e.g. bHLH051, TMO5) from the basal cell lineage. Future experiments would be required to test whether such basal cell lineage enriched and depleted bHLH TFs respectively activate and repress genes sets characteristic of the basal cell lineage, but this is beyond the scope of the current study.

Details:

168 and 212: “Cluster 9 had mixed cell type assignments” & “clusters 1, 5 and 9 represent dividing subprotoderm”. Does this mean that dividing cells in the embryo proper and suspensors tend to lose their cell-fate markers?

We wrote “...our results suggested that clusters 1, 5 and 9 represent dividing subprotoderm (dividing inner; div.i), protoderm (dividing outer; div.o) and dividing cells in general (div), respectively (Fig. 2G).” Based on our in silico and in situ analyses, we think cluster 9 represents dividing cells in general.

188: “AT1G044880” – is this “AT1G04880”, as in Fig. 2F?

Thank you for spotting our error. We have corrected this typo in the revised manuscript.

214: “Altogether, our in silico and in situ validations indicated that. we can assign […] libraries to the major cell types.” It would be good if the authors could clarify minor discrepancies between results and expectations. Three clusters corresponding to “suspensor” were found – why, are there really three different cell types in the suspensor? No clusters corresponding to “upper inner periphery” and “upper protoderm” were found – why, not enough nuclei?

We believe the discrepancies between results and expectations were due to sampling issues and the nature of the nine expected groups. Our study had a relatively small sample size (486 snRNA-seq were retained after stringent quality controls), which does not favor resolving rare cell types. Indeed, the relatively large number of suspensor, columella initial and quiescent center clusters indicated that there was sampling bias when collecting embryonic nuclei, as releasing nuclei from the filamentous basal cell lineage is easier than releasing nuclei from internal layers of the embryo proper (e.g. upper inner periphery). This sampling bias likely contributes to why there are only a few upper inner periphery nuclei in our dataset, which would make it impossible to form a distinct cluster. In addition, the missing upper inner periphery and ground tissue initials have less specific markers reported in the literature compared to other cell types, making them more difficult to detect with our marker-based approach. These inner cell types might also have transitional fates in the early globular embryos we collected, as the nine cell types were conventionally defined in mid or late globular embryos. Increasing the number of snRNA-seq libraries may compensate for sampling biases, but this may also be due to the transient fates of these emerging embryonic cell types as we attempted to clarify in the revised text. Regarding the three suspensor clusters, it is common that multiple clusters can have the same cell type assignment and our ISH results indicated that these three clusters represent suspensors as described in the text.

© 2021. Published by The Company of Biologists under the terms of the Creative Commons Attribution License (https://creativecommons.org/licenses/by/4.0/).

224: “13,893 transcripts detected in ≥10% of nuclei in ≥1 cluster”. Is it possible to be in ≥10% of nuclei in <1 cluster?

Yes it is possible for a gene to be expressed in ≥10% of all nuclei, but not concentrated in any cluster. However, we would not have retained these genes for downstream analyses because they were below the cut-off we used. For example, cluster 11 consists of 51 nuclei a gene’s transcripts had to be detected in at least 6 nuclei to be included. We clarified this in the revised text.

287 & 303: expression of CLSY1, NRPD1A, DCL3, ROS1, DML2, DML3. It is interesting finding that these genes are enriched in the basal cell lineage, but no validation is presented. ISH would be ideal but maybe too much of an effort; could published profiles be consulted?

We reasoned that by first validating the identities of the 12 clusters (Fig. 2), then we could study the expression patterns of selected genes with confidence. However, it is a good idea to confirm with published profiles for CLSY1, NRPD1A and DCL3 like we did for ROS1, DML2 and DML3 in Fig. 4C. We consulted published ACL/embryo proper and BCL/suspensor and indeed all of these genes were preferentially expressed in globular BCLs (Belmonte, 2013; Zhou, 2020). We have added this confirmation to the revised manuscript.

287 and elsewhere: “terminally differentiated suspensor”. We are not sure it is fair to say that the suspensor cells of globular embryos are terminally differentiated. For example, the scatter plots do not seem to suggest that the nuclei were polyploid yet.

Thank you, but we think that “terminally differentiated” is an appropriate term for suspensors because they stop dividing, undergo programmed cell death and do not contribute to post-embryonic tissues. This term is commonly used in the field to describe Arabidopsis suspenders and does not necessarily imply polyploidy although the suspensors of some plant species are polyploid.

Figure 3A – enriched GO terms. Microsporocyte differentiation is an unexpected category. Any explanation?

We agree that “microsporocyte differentiation” was an unexpected GO term enrichment for vascular initials. In the Panther database there are only two Arabidopsis genes with the “microsporocyte differentiation” GO term and they are the CLV1-related receptor-like kinases BARELY ANY MERISTEM1/2 (BAM1/2). BAM1/2 are required for leaf vascular patterning (DeYoung et al. 2005 PlantJ) and very recently were shown to be required for root xylem patterning (Fan et al. 2021 PNAS). Both BAM1 and BAM2 are in the “vas” top-ranked genes and this is the reason this GO term shows up as significant.

Fig. S2: “C” in the legend seems to correspond to “D” in the figure?

Thank you for pointing out this error. We have corrected it in the revised supplementary information document.

The recent paper by Farmer et al. should be added to the references (doi: 10.1016/j.molp.2021.01.001).

We had missed citing this recently published paper but have added the citation in the revised manuscript.

Reviewer 3 Advance summary and potential significance to field...
In this manuscript by Kao et al., the author present what I believe is the first data on single cell sequencing of Arabidopsis globular stage embryos. The authors use a pWOX2::H2B-GFP transgenic line in order to isolate nuclei specifically from the developing embryo and not the surrounding maternal tissue and endosperm. The authors then isolated RNA from these nuclei and perform several quality control measure and ultimately generated 486 single cell libraries. Library sequences were binned according to the co-expression of known markers for the embryo and were able to make informed predictions as to where multiple new genes would be

© 2021. Published by The Company of Biologists under the terms of the Creative Commons Attribution License (https://creativecommons.org/licenses/by/4.0/).

expressed. The authors show a nice correlation between their cell type predictions by presenting high quality in situ hybridization data in figure 2. They were clearly able to distinguish between RNAs found in the suspensor vs the embryo proper as well as subsets of cell types such as the protoderm. They then present an analysis of epigenetic regulators and their preferential accumulation pattern which is interesting. A final analysis of transcription factor binding motifs is also presented.

Overall, the data appears of high quality and the Nodine group is known for their work on early embryonic gene expression and have shown they have the technical skills required to perform these types of analyses that are embryo specific and not contaminated with other tissue. The datasets presented will be a valuable resource for those working on this stage of embryogenesis, although overall the paper feels a bit thin in comparison to recently published single cell RNA Seq papers that are being published at a rapid pace. The confirmation of cell type expression is nice, but really no further functional data is presented.

Thank you for the overall positive comments. Although we agree that additional experiments yielding insights into cell-specific mechanisms are exciting future research directions, we think they are beyond the scope of our current study on a developmentally relevant and intrinsically difficult tissue. Our goal was to provide a single-cell mRNA-seq method and associated gene expression atlas for early plant embryos.

Reviewer 3 Comments for the author...
My main concern is the final analysis in the section entitled “Differential enrichment of transcription factor binding motifs”. I am not sure this really adds much to the paper and seems highly speculative, especially the last section on the co-occurance of ERF binding sites and teloboxes. This last example could be deleted without affecting the take homes of the manuscript.

Thank you for this constructive comment. We originally included the section on co-occurrence of ERF and TBP binding motifs because we thought this was a significant and exciting result. Nevertheless, we understand and appreciate this comment which is also consistent with one of Reviewer #2’s comments. Therefore, we have removed the corresponding section from the revised manuscript and removed Fig. 5D-G and Fig. 6.

<u>Second decision letter</u>

MS ID#: DEVELOP/2021/199589

MS TITLE: Gene Expression Variation in Arabidopsis Embryos at Single-Nucleus Resolution

AUTHORS: Ping Kao, Michael A Schon, Magdalena Mosiolek, Balaji Enugutti, and Michael D Nodine
ARTICLE TYPE: Research Article

I am happy to tell you that your manuscript has been accepted for publication in Development, pending our standard ethics checks.

<u>Reviewer 1</u>

Advance summary and potential significance to field

The authors answered the previous comments and together with the changes in response to the comments of the two other reviewers, I believe the manuscript reads much better, is less speculative in its last part, and will provide valuable resources for the community.

Comments for the author

© 2021. Published by The Company of Biologists under the terms of the Creative Commons Attribution License (https://creativecommons.org/licenses/by/4.0/).

I am not apt to comment on the quality of the bioinformatic analysis (as you probably noticed from the initial review).
I am satisfied with the current version. But still, I have three remarks.
1- The resolution of the text with the formulas in the method (lines 632-641) is pretty bad. There is probably an option in Word office to insert formulas that will be a proper reading resolution 2- Line 510, please describe the method used for the embryonic phenotype observation of the rdd triple mutant. Nobody likes to have to dig into references to eventually find the referred methods. It takes two lines:
chloral hydrate clearing, microscopy observations with DIC optics, 40x objectives(?), n embryos out of x siliques, from x plants. What WT control used? Seeds obtained from?
3- In the supplementals, in legends for S2 and S3, second page, there is a copy-paste mistake, "Figure S4 (continued)". It should be Figure S2 on page 3 and S3 on page 5.

<u>Reviewer 2</u>

Advance summary and potential significance to field

This is the revised version of a report on RNA-sequencing of single nuclei prepared form globular-stage Arabidopsis embryos.

Comments for the author

The original version of this manuscripts met with overall very positive reviews. A number of relatively minor changes were made in the revisions, mostly small additions aimed at clarifying details. Also, a somewhat more speculative section on potential co-operative or competitive interactions between predicted transcription factor binding sites was eliminated, making the revised manuscript a bit more concise. I believe all the reviewer's comments have been adequately addressed, this seems ready to go. Congratulations to the authors for a rather nice piece of work.

© 2021. Published by The Company of Biologists under the terms of the Creative Commons Attribution License (https://creativecommons.org/licenses/by/4.0/).
